# Glucose-6-Phosphatase-Dehydrogenase activity as modulative association between Parkinson’s disease and periodontitis

**DOI:** 10.3389/fcimb.2024.1298546

**Published:** 2024-02-07

**Authors:** Oliver Laugisch, Marina C. Ruppert-Jungck, Thorsten M. Auschill, Sigrun Eick, Anton Sculean, Christian Heumann, Lars Timmermann, David J. Pedrosa, Carsten Eggers, Nicole B. Arweiler

**Affiliations:** ^1^ Department of Periodontology and Peri-Implant Diseases, Universitätsklinikum Giessen und Marburg (UKGM), Philipps University, Marburg, Germany; ^2^ Department of Neurology, University Hospital Giessen and Marburg, Marburg, Germany; ^3^ Center for Mind, Brain and Behavior (CMBB), Universities of Giessen and Marburg, Marburg, Germany; ^4^ Department of Periodontology, School of Dental Medicine, University of Bern, Bern, Switzerland; ^5^ Department of Statistics, Ludwig-Maximilians-University of Munich, Munich, Germany; ^6^ Department of Neurology, Knappschaftskrankenhaus Bottrop, Bottrop, Germany

**Keywords:** Parkinson’s disease, periodontal disease, neuroinflammation, periodontal pathogens, G6PD

## Abstract

The association between periodontitis (PD) and Parkinson’s disease (PK) is discussed due to the inflammatory component of neurodegenerative processes. PK severity and affected areas were determined using the following neuropsychological tests: Unified Parkinson’s Disease Rating Score (UPDRS) and Hoehn and Yahr; non-motoric symptoms by Non-Motor Symptoms Scale (NMSS), and cognitive involvement by Mini-Mental State Examination (MMSE). Neuroinflammation and the resulting Glucose-6-Phosphatase-Dehydrogenase (G6PD) dysfunction are part of the pathophysiology of PK. This study aimed to evaluate these associations in periodontal inflammation. Clinical data and saliva-, serum-, and RNA-biobank samples of 50 well-characterized diametric patients with PK and five age- and sex-matched neurologically healthy participants were analyzed for G6PD function, periodontal pathogens (*Aggregatibacter actinomycetemcomitans, Porphyromonas gingivalis, Tannerella forsythia, Treponema denticola, Prevotella intermedia, Campylobacter rectus, Fusobacterium nucleatum*, and *Filifactor alocis)*, monocyte chemoattractant protein (MCP) 1, and interleukin (IL) 1-beta. Regression analysis was used to identify associations between clinical and behavioral data, and t-tests were used to compare health and disease. Compared with PK, no pathogens and lower inflammatory markers (p < 0.001) were detectible in healthy saliva and serum, PK-severity/UPDRS interrelated with the occurrence of *Prevotella intermedia* in serum as well as IL1-beta levels in serum and saliva (p = 0.006, 0.019, 0.034), Hoehn and Yahr correlated with *Porphyromonas gingivalis*, *Prevotella intermedia*, RNA IL1-beta regulation, serum, and saliva IL1-beta levels, with p-values of 0.038, 0.011, 0.008, <0.001, and 0.010, while MMSE was associated with *Aggregatibacter actinomycetemcomitans*, *Fusobacterium nucleatum*, serum MCP 1 levels, RNA IL1-beta regulation and G6PD serum activity (p = 0.036, 0.003, 0.045, <0.001, and 0.021). Cognitive and motor skills seem to be important as representative tests are associated with periodontal pathogens and oral/general inflammation, wherein G6PD-saliva dysfunction might be involved.

**Clinical trial registration:**

https://www.bfarm.de/DE/Das-BfArM/Aufgaben/Deutsches-Register-Klinischer-Studien/_node.html, identifier DRKS00005388.

## Introduction

Periodontitis (PD) is an inflammatory disease that leads to the loss of tooth-supporting structures caused by the host’s response to periodontal pathogens (Page and Kornman, 1997). *Porphyromonas gingivalis*, *Aggregatibacter actinomycetemcommitans*, *Tannerella forsythia*, and *Treponema denticula* are the most common periodontal pathogens, whereas *Campylobacter rectus* is associated with chronic PD and a number of systemic conditions, such as prematurity (Madianos et al., 2001), preterm low birth weight (Buduneli et al., 2005), and arteriosclerosis (Fiehn et al., 2005). *Fusobacterium nucleatum* is known to bridge the gap between early and late colonizers (Kolenbrander et al., 2002). Elevated levels of anti-pathogen antibodies in cerebro-spinal-fluid highlight the possibility of an intrathecal immune response (Laugisch et al., 2018).

The second most prevalent neurodegenerative disorder, Parkinson’s disease (PK), is caused by the specific degeneration of dopaminergic neurons, predominantly affecting the lateral tier of the *Substantia Nigra* (Schulte et al., 2016). While the key neuropathological hallmarks, including the loss of dopaminergic neurons in the *Substantia Nigra*, correspond well with the observed motor complications, little is known about the initial cause of neural cell degeneration, and sufficient explanations for the complexity of the disease are lacking. The disease profile of PK is often very complex, with the additional involvement of autonomic, sensory, and cognitive deficits or their combinations, partly occurring in prodromal disease phases. Neurological and neuropsychological tests help classify PK and its severity. The Hoehn & Yahr Index (Hoehn and Yahr, 1967) classifies severity ranging from stage 0 (no signs of disease) to 5 (without help bound to a wheelchair or bedridden), and the Unified Parkinson’s Disease Rating Scale (UPDRS) III is a motoric examination with a 0 – 52 scale representing the most severe motor involvement (Goetz et al., 2008). Patients suffering from non-motoric symptoms in the previous month were assessed using the Non-Motor Symptoms Scale (NMSS) (van Wamelen et al., 2021).

Over the last decade, neuroinflammation has gained prominence as a key factor in the pathogenesis of PK (More et al., 2013; Nolan et al., 2013; Vivekanantham et al., 2015) and oxidative stress is of great interest, especially via the pentose-phosphate pathway, as bacterial lipopolysaccharides (LPS) are associated with glucose-6-phosphate dehydrogenase (G6PD) dysfunction (Tu et al., 2019).

While PD and PK share the same risk factors, increased frequencies of PD have been shown in long-term PK when compared to age- and bias-matched groups. Modulative associations between systemic diseases (e.g., diabetes, rheumatoid arthritis, and Alzheimer’s disease) and PD have been described in the literature (Laugisch et al., 2016; Laugisch et al., 2018).

More recently, research was conducted to investigate the missing link between PD and PK (Kaur et al., 2016). Early attempts were based on a report demonstrating that patients with PK experience hyposalivation and xerostomia, leading to an impaired oral health-related quality of life (Barbe et al., 2017). Chronic inflammatory responses such as the release of interleukin-1 (IL-1), interleukin-6 (IL-6), tumor-necrosis factor-alpha (TNF-α), all characteristic for PD (Ebersole and Cappelli, 2000), might be one of the etiological causes of PK (Wu et al., 2016).

A large nationwide population-based case-control study based on health insurance data in Taiwan showed that periodontal inflammation is associated with the risk of PK (Chen et al., 2017) and previous periodontal treatment substantially lowers the risk of later development (Chen et al., 2018). Although these studies from one workgroup had several limitations, they were the only ones that confirmed an association between PD and early onset PK.

Thus, the aim of this retrospective study was to examine biobank blood and saliva samples for inflammatory biomarkers and periodontal pathogens to systemically evaluate the putative relationship between PK and signs of PD.

It was hypothesized that:**1**) more periodontal pathogens are detectible in the saliva of patients with PK than in healthy patients; **2**) patients with PK in relation to healthy controls suffer from inflammation measured by monocyte chemoattractant protein (MCP) 1 and IL 1-beta levels in saliva and serum; **3**) PK is associated with G6PD dysfunction compared to healthy individuals; **4**) PK severity correlates with the amount of periodontal pathogens in saliva, inflammation measured by MCP1 and IL-1-beta levels in serum and saliva as well as an impaired G6PD-function; **5**) among the patients severely diseased with PK, more periodontal pathogens in saliva result in the presence of inflammatory biomarkers and a lower G6PD activity both in serum and saliva.

## Methods

### Sample size calculation

The sample size calculation revealed that 50 PK samples were required for inclusion. The necessary sample size was evaluated using probit analysis simulations to estimate the limit of detection (LoD) when the samples were measured in duplicate. A width of the 95% confidence interval (CI) of about 0.5 units can be established, when a sample size of 50 is used. Additionally, the only available five non-PK patients with all the necessary parameters were included.

### Study center, subjects and samples

From a larger, well-characterized cohort (KFO 219), saliva, RNA, and serum samples were collected from 50 patients with PK and 5 healthy controls. Fifty patients diagnosed with idiopathic PK were recruited from the Department of Neurology of University Hospital Cologne in collaboration with community neurologists. Diagnoses were made according to the UK Brain-Bank criteria (Gibb and Lees, 1988) by an experienced movement disorder specialist. Additionally, biobank samples from five age- and sex-matched, neurologically healthy controls were included. Patients underwent an extensive study protocol involving the assessment of motor, cognitive, neuropsychiatric, and other non-motor symptoms; multimodal neuroimaging; and metabolomic and genetic analyses. All procedures have been described previously (Eggers et al., 2011; Glaab et al., 2019; Hammes et al., 2019; Ruppert et al., 2020; Ruppert et al., 2021). The inclusion criteria were as follows: age > 40 years and absence of dementia or conditions directly impairing brain function. In the PK group, Hoehn and Yahr stages I-III and tolerability of the complete medical OFF state, defined as a minimum of 12 h without levodopa, amantadine, and monoamine oxidase-inhibitors and 72 h discontinuation of dopamine agonists, were required. Moreover, suspected atypical Parkinsonian syndromes, advanced Parkinsonism [Hoehn and Yahr stage >3 (Hoehn and Yahr, 1967), dementia, CNS diseases other than PK, and any safety concerns regarding MRI prohibited study inclusion. Patients with dementia were excluded according to the criteria published by the Movement Disorder Society using a neuropsychological test battery and assessment of the patient’s ability to manage daily life (Emre et al., 2007).

### Clinical examination

The available data included age, sex, weight, height, years of education, and PK duration. Global cognitive screening was performed using the Mini-Mental State Examination (MMSE) (Folstein et al., 1975). Functional, anatomical, and clinical data were collected at the Max Planck Institute for Metabolism Research and the Department of Neurology, University Hospital Cologne (UHC). The levodopa-equivalent daily dose (LEDD) was calculated for all antiparkinsonian medications and separately for dopamine agonists based on standard conventions (Tomlinson et al., 2010). Clinical examination was performed in the OFF state, defined as a 12-h period without dopaminergic medication(Langston et al., 1992) (72 h in cases of dopamine agonists). A few neurological and neuropsychological tests were performed to classify PK and its motor and non-motor symptom severity and were applied in the current study. The Hoehn & Yahr Index (Hoehn and Yahr, 1967) classifies severity ranging from stage 0 (no signs of disease) to 5 (without help bound to a wheelchair or bedridden), and the UPDRS III is a motoric examination with a 0 – 52 scale representing the most severe motor involvement (Goetz et al., 2008). Patients suffering from non-motoric symptoms in the last month, non-motor symptoms were assessed using the Non-Motor Symptoms Scale NMSS (van Wamelen et al., 2021).

### Blood and saliva collection and RNA processing

Blood samples were collected at the UHC in the morning. Venipuncture sets of the “butterfly” type (Venofix A, 0.8mm or 0,65mm (Braun, Germany) were used with K2-EDTA vacutainers. Samples were visually checked for hemolysis, which would result in red-coloring of the samples due to increased free hemoglobin concentrations, but no hemolyzed sample was detected. Venous blood was drawn from the cubital vein in a 10 ml EDTA vacuum tube. Immediately after collection, tubes were inverted 8 times and placed on ice. Processing at UHC was performed by the same person for all subjects. Samples were centrifuged at 2000×*g* for 10min at +4°C to separate the plasma, which was then homogenized by pipetting, divided into 10 matrix tubes of 500 μl each and stored at −80°C. For transfer to the Integrated Biobank of Luxemburg (IBBL) the matrix tubes were packed in dry ice. At IBBL, RNA was isolated automatically from EDTA blood using PAXgene (Qiagen, Venlo, The Netherlands).

For saliva sampling, participants were not allowed to eat, drink or smoke 1-hour prior sample collection. Five minutes ahead, they had to rinse their mouth with tap water, wash their hands and clean them with a clean towel. 4ml unstimulated Saliva (depending on saliva flow-rate) is needed to be spat into a Salimetrics collection device (Greiner Bio One) excluding any bubbles for 3 to 5 minutes. Processing at UHC was performed by the same person for all subjects. After centrifugation, the sample was divided into 10 matrix tubes of 400 μl each and stored at −80°C. For transfer to the Integrated Biobank of Luxemburg (IBBL) the matrix tubes were packed in dry ice.

### Biospecimen procedure

After shipping on dry ice, saliva samples were analyzed for presence of periodontal pathogens, inflammation (IL-1beta, MCP-1/CCL-2) and G6PD-activity, serum was investigated for inflammatory biomarkers and G6PD-activity, while in RNA Levels of IL-1beta, MCP-1/CCL-2 and G6PD expression were determined.

### Determination of selected microorganisms and inflammatory mediators

DNA was extracted from saliva using a commercial kit (High Pure Template Preparation Kit; Roche, Basel, Switzerland) according to the manufacturer’s instructions for bacterial DNA. Counts presented as log10 of *A. actinomycetemcomitans, P. gingivalis, T. forsythia Treponema denticola, P. intermedia, C. rectus, F. nucleatum*, and *F. alocis* were determined using real-time polymerase chain reaction (qPCR), as previously described (Jentsch et al., 2017; Eick et al., 2018; Laugisch et al., 2018). The counts are presented as log 10. The levels of IL-1 and MCP-1/CCL-2 in the saliva and serum samples were determined using commercially available ELISA kits (R&D Systems Europe Ltd., Abingdon, UK), according to the manufacturer’s instructions. The detection level of these kits was 1 pg/ml in serum or 1 pg/μL saliva.

### Determination of Glycose-6-Phosphate Dehydrogenase activity

In saliva and serum, enzyme activity was determined in milliunits/ml using the MAK015 Glucose-6-Phosphate Dehydrogenase Assay Kit (Sigma-Aldrich, St. Louis, USA) according to the manufacturer’s instructions.

### mRNA analysis

The RNA samples were transcribed in cDNA using the GoSrcipt™ Reverse Transcription System (Promega, Madison, USA) according manufacturer`s description. Levels of IL-1β, MCP-1/CCL-2 and G6PD expression as relative quantity were determined by a following qPCR using GoTaq^®^ qPRC mastermix (Promega) with respected primers designed according to the database http://www.oralgen.lanl.gov (IL-1β, forward: 5′-TAC GAA TCT CCG ACC ACCACT ACA G-3 and reverse: 5′-TGG AGG TGG AGA GCT TTC AGT TCA TAT G-3′; MCP-1, forward: 5′-GAT CTC AGT GCA GAG GCT CG-3′ and reverse: 5′-TTT GCT TGT CCA GGT GGT CC-3′; G6PD, forward: 5′-CTG TTC CGT GAG GAC CAG ATC T-3′ and reverse; 5′-TGA AGG TGA GGA TAA CGC AGG C-3′) and were calculated by means of the comparative delta-DCt method (Livak and Schmittgen, 2001).

### Clinical data

#### Data management/data collection forms

The biosample dataset was merged with the available clinical data, pseudonymized, and stored in Excel sheets (Redmond, WA, USA).

### Statistical analysis

All statistical analyses were performed using SPSS Version 23 (IBM, Armonk, NY, USA). Demographic and anamnestic data were described using interval scales, arithmetic means, standard deviations, minima, and maxima. For interferential statistics, non-parametric (Mann-Whitney, Person Chi-Quadrat) tests were used to compare the healthy and patients with PK. When healthy data (n=5) was compared with those of PK`s (n=50), different group sizes were taken into account.

Association analysis was performed using bivariate correlations (Spearman’s rank correlation) and partial correlation analysis after controlling for LEDD.

Linear regression analysis was performed for patients with PD using the UPDRS, Hoehn and Yahr, NMSS, and MMSE as dependent variables.

Age, sex, periodontal pathogens, inflammatory biomarkers, and enzyme activities were entered as covariates in the regression models of the demographic and biosample data.

A covariate was considered significant if the associated p-value in the regression model was lower than α = 0.05.

## Results

### General demographics

General demographic data such as age, weight, height, and years of education did not differ significantly between the groups (**Table 1**).

**Table 1:** Demographics in means + standard deviation (STD).

	Healthy	Parkinson`s	*Total*	p-value
N	5	50	55	>0.05
Mean Age	66.2 ( ± 8.7)	64.2 ( ± 9)	64.4 ( ± 8.9)	>0.05
Weight	69.2 (± 12.9)	81.7 (± 16.6)	80.5 ( ± 16.6)	>0.05
*Height*	172.0 ( ± 5.9)	175.5 ( ± 9.1)	175.2 ( ± 8.9)	>0.05
Years of education	15.8 ( ± 2.7)	14.1 ( ± 2.8)	14.2 (± 2.8)	>0.05

### Inter-group comparison

No periodontal pathogens were detected in the saliva of healthy individuals; however, they were detected in the saliva of all patients with PK. Highest mean log10 frequencies were determined for *F. nucleatum* 4.7 (± 1.3) followed by *P. intermedia, C. rectus*, and *P. gingivalis with* 2.3 (±2.6), 2.1 (± 2.2), and 2.1 (± 2.2) respectively (**Table 2**). Additionally, bacterial complex building was observed in PK; for example, *P. gingivalis* and *T. forsythia* (p = 0.006) (**Supplementary Table 1**).

**Table 2:** Log10 frequencies of periodontal pathogens detectable in saliva.

	Healthy	Parkinson`s (mean ± STD)	*Parkinson`s (Min/Max)*
*A. actinomycetemcomitans*	0	1.1 ( ± 1.2)	0/5.0
*P. gingivalis*	0	2.1 ( ± 2.2)	0/5.0
*T. denticola*	0	1.5 ( ± 1.9)	0/4.8
*T. forsythia*	0	1.5 ( ± 2.2)	0/6.8
*C. rectus*	0	2.1 ( ± 2.2)	0/6.8
*F. alocis*	0	1.2 ( ± 2.3)	0/9.0
*F. nucleatum*	0	4.7 ( ± 1.3)	0/7.6
*P. intermedia*	0	2.3 ( ± 2.6)	0/7.6

MCP1/CCL2 and IL1-beta levels in the serum and saliva were significantly lower in healthy participants (p < 001^b^ and p = 001^b^//p < 001^b^ and 0.001^b^). However, the RNA upregulation of MCP1/CCL2 and IL-beta in PK was not significant (p = 0.244^b^ and p = 0.039^b^) (**Table 3**).

**Table 3:** Inflammatory markers and enzyme activity.

	Healthy (mean ± STD)	Healthy (Min/Max)	Parkinson`s (mean ± STD)	Parkinson`s (Min/Max)	p-value
RNA MCP1/CCL2	0.321 ± 0.106	0.164/0.432	0.328 ± 0.356	0.009/ 1.542	0.244^b^
Serum MCP1/CCL2	52.142 ± 28.584	12.280/81.880	547.63 ± 1.334.664	43.55/8,950.36	<0.001^b^
Saliva MCP1/CCL2	37.228 ± 10.091	30.3/55.1	247.01 ± 328.124	28.21/1,519.43	<0.001^b^
RNA IL1-beta	0.680 ± 0.334	0.351/1.183	2.040 ± 4.052	0.038/28.527	0.039^b^
Serum IL1-beta	14.588 ± 6.108	9.76/24.72	128.007 ± 221.653	6.34/ 978.00	0.001^b^
Saliva IL1-beta	57.618 ± 26.225	28.89/76.77	949.429 ± 1,120.875	12.82/5,034.00	0.001^b^
RNA G6PD	0.907 ± 0.224	0.563/1.157	1.150 ± 0.321	0.476/ 1.193	0.093^b^
Serum G6PD	14.96 ± 3.23	10.9/19.6	4.628 ± 2.495	0/ 12.0	<0.001^b^
Saliva G6PD	12.380 ± 1.429	11.1/14.8	1.148 ± 4.759	0/ 33.9	<0.001^b^

^b^Exact two-sided p-values of a Mann-Whitney-U-Test for two independent samples.

Concerning G6PD in saliva and serum, PK showed significantly lower activity values, that is, 1.148 ± 4.759 units in saliva vs. 12.380 ± 1.429 units in ‘healthy’ saliva (p = <0.001^b^) as well as in serum:4.628 ± 2.495 units vs. 14.96 ± 3.23 units (p < 0.001^b^) although their RNA regulation was not (p = 0.093^b)^ (**Table 3**).

### G6PD-activity in association with periodontal pathogens

Lower enzyme activity was statistically associated with all pathogens in saliva, but only with *P. gingivalis, T. denticola, C. rectus, F. alocis, F. nucleatum, and P. intermedia* in serum, and was upregulated when *P. gingivalis, T. denticola, and T. forsythia* was determined (**Supplementary Table 2**).

### Clinical and behavioral data

Clinical test results, including motor severity quantified by UPDRS-III and Hoehn and Yahr scores, as well as NMSS scores, differed between healthy participants and patients with Parkinson’s disease, whereas cognitive performance in the MMSE was comparable (**Table 4**). Bivariate and partial Correlations with measures of oral inflammation and periodontal pathogens, including LEDD as covariates, are shown in **Supplementary Tables 3**, **4**.

**Table 4:** Clinical and behavioral data.

	Healthy	Parkinson`s
UPDRS	0	26.51
Hoehn & Yahr	0	2.43
NMSS	10.8	55.54
Mini-Mental-State	29.6	28.48
LEDD	0	491.77

### Inter-Parkinson group comparison

PK severity measured by UPDRS-III was significantly associated with *Prevotella intermedia*, serum IL1beta levels, saliva IL1-beta levels and years of education (p = 0.006, 0.019, 0.034 and 0.005 respectively) (**Table 5**), while Hoehn &Yahr correlated with *Porphyromonas gingivalis*, *Prevotella intermedia*, RNA IL1-beta regulation, serum IL1-beta levels, saliva IL1-beta levels and duration of Parkinson`s disease with p-values of 0.038, 0.011, 0.008, <0.001, 0.010, and 0.001 (**Table 6**). The non-motor scale showed no statistically significant associations (**Table 7**), whereas cognitive evaluation using the MMSE was associated with *A. actinomycetemcomitans*, *Fusobacterium nucleatum*, serum MCP 1 levels, RNA IL1-beta regulation, G6PD Serum activity, and years of education (p=0.036, 0.003, 0.045, <0.001, 0.021, and 0.072, respectively) (**Table 8**).

**Table 5:** Regression model of variables dependent on UPDRS.

	Estimate	Std.-error	95% CI				
			lower	upper	Chi-square	df	Sig.
(constant)	46.610	16.4506	14.367	78.852	8.028	1	0.005
[gender=male]	1.691	3.4246	-5.021	8.403	0.244	1	0.621
[gender=female]	0^a^						
A.a. log 10	0.856	0.7696	-0.653	2.364	1.236	1	0.266
P.g. log 10	0.794	0.5952	-0.373	1.960	1.778	1	0.182
T.d. log 10	0.400	0.9380	-1.439	2.238	0.182	1	0.670
T.f. log 10	-0.362	0.6629	-1.662	0.937	0.299	1	0.585
C.r. log 10	1.369	0.8932	-0.382	3.120	2.350	1	0.125
F.a. log 10	-0.104	0.7634	-1.600	1.392	0.019	1	0.891
F.n. log 10	-0.895	1.3105	-3.464	1.673	0.467	1	0.495
P.i. log 10	1.649	0.6038	0.466	2.833	7.460	1	0.006
RNA MCP1	2.506	4.9837	-7.262	12.274	0.253	1	0.615
Serum MCP 1	0.000	0.0009	-0.001	0.002	0.135	1	0.713
Saliva MCP1	0.006	0.0046	-0.003	0.015	1.588	1	0.208
RNA IL1-beta	-0.154	0.3272	-0.795	0.487	0.221	1	0.638
Serum IL1-beta	0.015	0.0064	0.002	0.027	5.476	1	0.019
Saliva IL1-beta	-0.004	0.0017	-0.007	0.000	4.489	1	0.034
RNA G6PD	-5.741	5.6417	-16.799	5.316	1.036	1	0.309
G6PD Serum	-0.424	0.5047	-1.413	0.566	0.704	1	0.401
G6PD Saliva	0.376	0.2418	-0.097	0.850	2.424	1	0.120
LEDD	0.006	0.0041	-0.002	0.014	2.166	1	0.141
Age	-0.029	0.1610	-0.345	0.286	0.033	1	0.855
Years of education	-1.559	0.5508	-2.639	-0.480	8.012	1	0.005
Duration of PK	0.415	0.3992	-0.367	1.198	1.081	1	0.298
(Scale)	46.025^b^	9.2985	30.976	68.386			

^a^Coefficient is set to zero as this parameter is redundant and not estimated (dummy coding of the variable gender, male is coded as 1, female as 0).

^b^Scale parameter is estimated by the maximum likelihood method.

**Table 6:** Regression model of variables dependent on Hoehn&Yahr.

	Estimate	Std.-error	95% CI				
			lower	upper	Chi-square	df	Sig.
(constant)	2.540	0.7456	1.079	4.002	11.608	1	0.001
[gender=male]	-0.379	0.1552	-0.683	-0.074	5.950	1	0.015
[gender=female]	0^a^						
A.a. log 10	-0.023	0.0349	-0.091	0.045	0.429	1	0.512
P.g. log 10	0.054	0.0262	0.003	0.106	4.313	1	0.038
T.d. log 10	-0.027	0.0423	-0.110	0.056	0.404	1	0.525
T.f. log 10	0.023	0.0289	-0.034	0.080	0.637	1	0.425
C.r. log 10	0.062	0.0406	-0.017	0.142	2.349	1	0.125
F.a. log 10	-0.022	0.0342	-0.089	0.045	0.408	1	0.523
F.n. log 10	0.097	0.0565	-0.014	0.208	2.945	1	0.086
P.i. log 10	0.067	0.0264	0.015	0.119	6.410	1	0.011
RNA MCP1	-0.013	0.2247	-0.454	0.427	0.003	1	0.953
Serum MCP 1	0.000	0.000	0.000	0.000	1.892	1	0.169
Saliva MCP1	0.000	0.0002	-0.001	0.000	1.475	1	0.224
RNA IL1-beta	-0.039	0.0149	-0.068	-0.010	6.948	1	0.008
Serum IL1-beta	0.001	0.0003	0.001	0.002	14.011	1	<0.001
Saliva IL1-beta	0.000	0.000	0.000	0.000	6.560	1	0.010
RNA G6PD	-0.474	0.2566	-0.976	0.029	3.406	1	0.065
G6PD Serum	0.004	0.0229	-0.041	0.049	0.029	1	0.866
G6PD Saliva	-0.008	0.0110	-0.029	0.014	0.468	1	0.494
LEDD	0.000	0.0002	0.000	0.000	0.167	1	0.682
age	-0.006	0.0073	-0.021	0.008	0.772	1	0.380
Years of education	0.010	0.0250	-0.039	0.059	0.152	1	0.697
Duration of PK	0.061	0.0179	0.026	0.097	11.777	1	0.001
(Scale)	.095^b^	0.0190	0.064	0.141			

^a^Coefficient is set to zero as this parameter is redundant and not estimated (dummy coding of the variable gender, male is coded as 1, female as 0).

^b^Scale parameter is estimated by the maximum likelihood method.

**Table 7:** Regression model of variables dependent on non-motor-scale.

	Estimate	Std.-Error	95% CI				
			lower	upper	Chi-square	df	Sig.
(constant)	186.197	89.8734	10.048	362.345	4.292	1	0.038
[gender=male]	-27.092	18.7098	-63.763	9.578	2.097	1	0.148
[gender=female]	0^a^						
A.a. log 10	1.472	4.2020	-6.763	9.708	0.123	1	0.726
P.g. log 10	5.014	3.1615	-1.183	11.210	2.515	1	0.113
T.d. log 10	6.104	5.0965	-3.885	16.093	1.434	1	0.231
T.f. log 10	3.094	3.4783	-3.723	9.912	0.791	1	0.374
C.r. log 10	6.134	4.8964	-3.462	15.731	1.570	1	0.210
F.a. log 10	-5.675	4.1217	-13.753	2.404	1.896	1	0.169
F.n. log 10	-4.178	6.8160	-17.537	9.181	0.376	1	0.540
P.i. log 10	1.025	3.1802	-5.209	7.258	0.104	1	0.747
RNA MCP1	30.058	27.0841	-23.026	83.142	1.232	1	0.267
Serum MCP 1	-0.002	0.0049	-0.011	0.008	0.146	1	0.702
Saliva MCP1	0.015	0.0254	-0.035	0.065	0.342	1	0.559
RNA IL1-beta	-1.097	1.7921	-4.610	2.415	0.375	1	0.540
Serum IL1-beta	0.034	0.0349	-0.035	0.102	0.940	1	0.332
Saliva IL1-beta	-0.002	0.0093	-0.020	0.017	0.034	1	0.854
RNA G6PD	-6.626	30.9271	-67.242	53.990	0.046	1	0.830
G6PD Serum	-0.756	2.7553	-6.156	4.645	0.075	1	0.784
G6PD Saliva	0.271	1.3228	-2.322	2.863	0.042	1	0.838
LEDD	0.010	0.0223	-0.033	0.054	0.213	1	0.645
age	-1.185	0.8798	-2.909	0.540	1.813	1	0.178
Years of education	-4.092	3.0150	-10.001	1.818	1.842	1	0.175
Duration of PK	-0.113	2.1592	-4.345	4.119	0.003	1	0.958
(Scale)	1383.494^b^	276.6989	934.840	2047.471			

^a^Coefficient is set to zero as this parameter is redundant and not estimated (dummy coding of the variable gender, male is coded as 1, female as 0).

^b^Scale parameter is estimated by the maximum likelihood method.

**Table 8:** Regression model of variables dependent on mini-mental state examination.

	Estimate	Std.-Error	95% CI				
			lower	upper	Chi-square	df	Sig.
(constant)	22.507	2.8783	16.866	28.149	61.148	1	<0.001
[gender=male]	0.912	0.5992	-0.263	2.086	2.315	1	0.128
[gender=female]	0^a^						
A.a. log 10	-0.282	0.1346	-0.545	-0.018	4.381	1	0.036
P.g. log 10	-0.007	0.1012	-0.205	0.191	0.005	1	0.945
T.d. log 10	-0.250	0.1632	-0.570	0.069	2.355	1	0.125
T.f. log 10	-0.255	0.1114	-0.473	-0.037	5.239	1	0.022
C.r. log 10	0.037	0.1568	-0.270	0.344	0.055	1	0.814
F.a. log 10	-0.111	0.1320	-0.370	0.147	0.710	1	0.399
F.n. log 10	0.655	0.2183	0.227	1.083	9.009	1	0.003
P.i. log 10	0.007	0.1018	-0.193	0.207	0.005	1	0.946
RNA MCP1	1.275	0.8674	-0.425	2.975	2.161	1	0.142
Serum MCP 1	0.000	0.0002	0.000	0.001	4.005	1	0.045
Saliva MCP1	0.000	0.0008	-0.002	0.002	0.004	1	0.952
RNA IL1-beta	-0.260	0.0574	-0.373	-0.148	20.576	1	<0.001
Serum IL1-beta	-0.001	0.0011	-0.003	0.001	0.693	1	0.405
Saliva IL1-beta	0.000	0.0003	-0.001	0.000	0.693	1	0.405
RNA G6PD	-0.616	0.9905	-2.557	1.325	0.387	1	0.534
G6PD Serum	0.203	0.0882	0.030	0.376	5.298	1	0.021
G6PD Saliva	-0.018	0.0424	-0.101	0.065	0.172	1	0.678
LEDD	0.000	0.0007	-0.001	0.002	0.237	1	0.627
age	0.014	0.0282	-0.041	0.070	0.261	1	0.610
Years of education	0.173	0.0966	-0.016	0.363	3.227	1	0.072
Duration of PK	-0.002	0.0692	-0.138	0.133	0.001	1	0.973
(Scale)	1.419^b^	0.2838	0.959	2.100			

^a^Coefficient is set to zero as this parameter is redundant and not estimated (dummy coding of the variable gender, male is coded as 1, female as 0).

^b^Scale parameter is estimated by the maximum likelihood method.

### Study hypotheses

This study confirmed our hypotheses as **1**) contrary to healthy patients, periodontal pathogens were detected in the saliva of patients with PK; **2**) in patients with PK, higher levels of MCP 1 and IL1-beta levels could be determined in both saliva and serum; **3**) G6PD dysfunction is observed in patients with PK compared to healthy individuals; **4**) PK severity correlated with the amount of periodontal pathogens in saliva, inflammation measured by MCP1 and IL-1-beta levels in serum and saliva as well as an impaired G6PD-function; **5**) at a more severe state of PK disease, more salivary periodontal pathogens result in presence of inflammatory biomarkers and a lower G6PD activity both in serum and saliva.

## Discussion

Within this retrospective analysis of biosamples gathered during a large PK study it could be shown that some periodontal pathogens were detectable in the saliva of patients with PK, but the same pathogens were not found in saliva of age- and gender-matched healthy individuals. Additionally, the severity of PK determined by Hoehn and Yahr and the UPDRS was associated with the burden of periodontal pathogens, as well as general and oral inflammation. Oral and general inflammation was significantly lower in the healthy group, whereas lower activity of G6PD was associated with PK. Motor and cognitive impairments were associated with inflammation and bacterial load, while non-motoric criteria showed no associations.

The concentration of periodontal pathogens in saliva is lower than that in subgingival plaque owing to their anaerobic characteristics, but a real-time PCR assay using saliva, as performed in this study, has the potential to evaluate periodontal health status, as described by Zhou et al. (2015). Considering the diversity in the individual/periodontal pathogenic microbiome, one should keep in mind, that samples were qPCR-tested just for eight pathogens in saliva obtained by using a saliva sampling technique that is very susceptible to errors. A dental/periodontal examination could have confirmed or refuted the true presence of PD but was not the focus of the collection of biobank samples.

Also, it was a surprise for the working group to find this significant difference between the PK group and healthy controls. The small number of 5 healthy controls versus 50 participants in the PK group is surely a critical point. When scanning the biobank, finally there were only 5 healthy controls left that matched to those of the test group. However, in both groups’ qPCR was performed in exactly the same way and moreover, the statistical analysis took the different group sizes into account.

So far, this study is the only one to investigate periodontal pathogens in patients with PK. An analysis of PK patients from an extensively characterized cohort in terms of clinical and neuropsychological test results, and therefore affected brain areas, is a strength.

Several studies have investigated oral health in neurodegenerative diseases; however, as this field is very complex, a closer look at the details has not been conducted until now. A battery of clinical and neuropsychological tests was used to exclude cognitive impairment (healthy controls and patients with PK had an MMSE score of approximately 30 after screening healthy:29.6, PK:28.48), to determine disease severity, and to distinguish between motoric and non-motoric involvement. This study verified a bidirectional relationship between PK severity, motoric/cognitive involvement, and periodontal pathogens in saliva samples. Several other studies have reported that fine motor impairments and cognitive decline in PK complicate oral hygiene habits, resulting in poor oral health status; however, none of these studies considered the entire spectrum of symptoms. The studies from Hanaoka and van Stiphoud are the only ones that consider Hoehn & Yahr for determining PK severity (Hanaoka and Kashihara, 2009; van Stiphout et al., 2018), while a group from Brazil compared chewing with a new complete denture ‘on’ and ‘off-PK medication’ (Ribeiro et al., 2017), which should be interpreted with caution since habituation with a new complete denture is also a challenge for generally healthy individuals.

To the best of our knowledge, this is the first study to combine oral inflammation, G6PD activity and salivary levels of eight major periodontal pathogens in patients with PK. Interestingly, lower enzyme activity in the saliva was associated with all analyzed pathogens, and reduced activity in the serum was associated with all pathogens, except for *A. actinomycetemcomitans*. Consequently, the RNA coding for G6PD was significantly associated with four of the five most prevalent periodontal pathogens. P-values for the presence of *Porphyromonas gingivalis* were statistically significant in all PK biospecimens (serum, saliva, and RNA; −0.141, −0.141, and −0.103, respectively). This may indicate a special role for this known keystone pathogen, LPS, and gingipains in amplifying systemic inflammation for PK pathogenesis (Adams et al., 2019; Olsen et al., 2020; Jasemi et al., 2022; Lyra et al., 2022).

NHANES data have shown that periodontitis is associated with leukocytosis in PK patients (Botelho et al., 2020) underlining an inflammatory context as a well-accepted part of the pathology (Hirsch et al., 2005; Lee et al., 2009). Disturbed protein formation, mitochondrial function, and oxidative stress are involved in PK pathogenesis (Obeso et al., 2010). The Pentose-Phosphate-Pathway is the most ubiquitous metabolic pathway to gain the reducing agent Nicotinamide Adenine Dinucleotide Phosphate (commonly known as NADPH), by the use of carbohydrates. NADPH is essential for maintaining a balanced Redox potential and activity of antioxidative enzymes within cells. In this context G6PD dysfunction is a missing source against antioxidative stress. Hence, a disrupted pathway is linked to the neuroinflammation involved in PK pathogenesis (Vivekanantham et al., 2015; Barbe et al., 2017; Tu et al., 2019). The aforementioned hints at a potential mechanistic background suggest that easily applicable chairside diagnostics for detecting G6PD activity in the saliva of patients with PK may be of great interest.

## Data availability statement

The original contributions presented in the study are included in the article/**Supplementary Material**. Further inquiries can be directed to the corresponding author.

## Ethics statement

The studies involving humans were approved by Local ethics committee of the Medical Faculty of the University of Cologne (number 12–165). The studies were conducted in accordance with the local legislation and institutional requirements. The participants provided their written informed consent to participate in this study.

## Author contributions

OL: Conceptualization, Data curation, Formal analysis, Funding acquisition, Investigation, Methodology, Project administration, Validation, Visualization, Writing – original draft, Writing – review & editing. MR: Data curation, Resources, Software, Validation, Writing – review & editing. TA: Conceptualization, Supervision, Validation, Writing – review & editing. SE: Resources, Supervision, Writing – review & editing. AS: Resources, Supervision, Writing – review & editing. CH: Data curation, Formal analysis, Resources, Software, Validation, Visualization, Writing – review & editing. LT: Conceptualization, Resources, Supervision, Validation, Writing – review & editing. DP: Supervision, Writing – review & editing. CE: Data curation, Resources, Supervision, Validation, Writing – review & editing. NA: Conceptualization, Project administration, Resources, Supervision, Validation, Writing – review & editing.
