# Supplementary material for: Glucose-6-Phosphatase-Dehydrogenase activity as modulative association between Parkinson’s disease and periodontitis
Source: Front Cell Infect Microbiol. 2024 Feb 7;14:1298546. doi: 10.3389/fcimb.2024.1298546 (PMC10885135; doi:10.3389/fcimb.2024.1298546)
Supplement: Supplementary file 1 [file Table_1.pdf]

# Glucose-6-Phosphatase-Dehydrogenase activity as modulative association between Parkinson`s disease and Periodontitis

Oliver Laugisch*[1], Ruppert-Jungck M[2], Auschill TM[1], Eick, S[3], Sculean A.[3], Heumann C[4], Timmermann L[2], Eggers C [2,5] and Arweiler NB[1]

[1] Department of Periodontology and Peri-Implant Diseases, Philipps University, Marburg, Germany

[2] Department of Neurology, University Hospital Giessen and Marburg, Marburg, Germany

[3] Department of Periodontology, School of Dental Medicine, University of Bern, Bern, Switzerland

[4] Department of Statistics, Ludwig-Maximilians-University Munich, Germany

[5] Center for Mind, Brain and Behavior (CMBB), Universities of Giessen and Marburg, Germany

*** Correspondence:**
Dr. Oliver Laugisch
Department of Periodontology and Peri-Implant Diseases
Philipps-University Marburg
Georg-Voigt-Straße 3
35039 Marburg
Tel.: +49 (0) 6421 58 63235
Fax: +49 (0) 6421 58 63270
Email: Oliver.Laugisch@uni-marburg.de

Running title: Parkinson`s and periodontal disease.

**SUPPLEMENTARY MATERIAL**

Table 1: Bivariate correlations

| | A.a. | P.g. | T.d. | T.f. | C.r. | F.a. | F.n. | P.i. |
|---|---|---|---|---|---|---|---|---|
| A.a. | | 0.325 | 0.634 | 0.001** | 0.169 | 0.698 | 0.188 | 0.210 |
| P.g. | 0.325 | | 0.913 | 0.006** | 0.824 | 0.648 | 0.601 | 0.791 |
| T.d. | 0.634 | 0.913 | | 0.118 | 0.000** | 0.000** | 0.000** | 0.141 |
| T.f. | 0.001** | 0.006** | 0.118 | | 0.576 | 0.668 | 0.526 | 0.980 |
| C.r. | 0.169 | 0.824 | 0.000** | 0.576 | | 0.000** | 0.001** | 0.040* |
| F.a. | 0.698 | 0.648 | 0.000** | 0.668 | 0.000** | | 0.000** | 0.047* |
| F.n. | 0.188 | 0.601 | 0.000** | 0.526 | 0.001** | 0.000** | | 0.159 |
| P.i. | 0.210 | 0.791 | 0.141 | 0.980 | 0.040* | 0.047 | 0.159 | |

**. The correlation is significant at the 0.01 level (two-sided)..
*. The correlation is significant at the 0.05 level (two-sided)..

Table 2: Bivariate correlations

| | UPDRS | Hoehn-Yahr. | **L-Dopa** | **Nms scale** | **mmst** |
|---|---|---|---|---|---|
| UPDRS | | 0.008** | 0.093 | 0.030 | 0.030 |
| Hoehn-Yahr | 0.008** | | 0.353 | 0.952 | 0.572 |
| L-Dopa | 0.093 | 0.353 | | 0.100 | 0.968 |
| **Nms** | 0.030* | 0.952 | 0.100 | | 0.172 |
| mmst | 0.030* | 0.572 | 0.968 | 0.172 | |

**. The correlation is significant at the 0.01 level (two-sided).

*. The correlation is significant at the 0.05 level (two-sided).

Table 3: Partial correlations dependent on L-dopa dose

| | UPDRS | Hoehn-Yahr. | Nms scale | mmst |
|---|---|---|---|---|
| UPDRS | | 0.000 | 0.002 | 0.002 |
| Hoehn-Yahr | 0.000 | | 0.020 | 0.482 |
| Nms scale | 0.002 | 0.020 | | 0.003 |
| mmst | 0.002 | 0.482 | 0.003 | |

Table 4: Partial correlations G6PD and Periodontal pathogens

| | **RNA** G6PD | **Serum G6PD** | ***Saliva G6PD*** |
|---|---|---|---|
| *A. actinomycetemcomitans* | 0.104 | 0.121 | -0.084 |
| *P. gingivalis* | -0.103 | -0.141 | -0.141 |
| *T. denticola* | -0.106 | -0.031 | -0.118 |
| *T. forsythia* | -0.049 | 0.065 | -0.109 |
| *C. rectus* | 0.155 | -0.070 | -0.153 |
| *F. alocis* | 0.289 | 0.050 | -0.073 |
| *F. nucleatum* | 0.173 | -0.126 | 0.006 |
| *P. intermedia* | 0,152 | -0,222 | -0,116 |
